# The brain-in-motion study: effect of a 6-month aerobic exercise intervention on cerebrovascular regulation and cognitive function in older adults

**DOI:** 10.1186/1471-2318-13-21

**Published:** 2013-02-28

**Authors:** Amanda V Tyndall, Margie H Davenport, Ben J Wilson, Grazyna M Burek, Genevieve Arsenault-Lapierre, Eryka Haley, Gail A Eskes, Christine M Friedenreich, Michael D Hill, David B Hogan, R Stewart Longman, Todd J Anderson, Richard Leigh, Eric E Smith, Marc J Poulin

**Affiliations:** 1 Department of Physiology & Pharmacology, Faculty of Medicine, University of Calgary, Calgary, Alberta T2N 4N1, Canada; 2 Hotchkiss Brain Institute, Faculty of Medicine, University of Calgary, Calgary, Alberta T2N 4N1, Canada; 3 Department of Medicine, Faculty of Medicine, University of Calgary, Calgary, Alberta, T2N 4N1, Canada; 4 Department of Clinical Neurosciences, Faculty of Medicine, University of Calgary, Calgary, Alberta, T2N 4N1, Canada; 5 Department of Psychiatry, Faculty of Medicine, Dalhousie University, Halifax, Nova Scotia, B3H 2E2, Canada; 6 Department of Psychology, Faculty of Science, Dalhousie University, Halifax, Nova Scotia, B3H 4R2, Canada; 7 Department of Medicine (Neurology), Dalhousie University, Halifax, Nova Scotia, B3H 3A7, Canada; 8 Department of Population Health Research, Alberta Health Services-Cancer Care, Calgary, Alberta, T2S 3C3, Canada; 9 Department of Oncology, Faculty of Medicine, University of Calgary, Calgary, Alberta, T2N 4N1, Canada; 10 Department of Community Health Sciences, Faculty of Medicine, Faculty of Medicine, University of Calgary, Calgary, Alberta, T2N 4N1, Canada; 11 Faculty of Kinesiology, University of Calgary, Calgary, Alberta, T2N 1N4, Canada; 12 Department of Radiology, Faculty of Medicine, University of Calgary, Calgary, Alberta, T2N 4N1, Canada; 13 Brenda Stafford Foundation Chair in Geriatric Medicine, Faculty of Medicine, University of Calgary, Calgary, Alberta, T2N 4N1, Canada; 14 Psychology Service, Alberta Health Services, Foothills Hospital, Calgary, Alberta, T2N 2T9, Canada; 15 University of Calgary, Calgary, Alberta, T2N 4N1, Canada; 16 Department of Cardiac Science, Faculty of Medicine, University of Calgary, Calgary, Alberta, T2N 4N1, Canada; 17 Airway Inflammation Research Group, Faculty of Medicine, University of Calgary, Calgary, Alberta, T2N 4N1, Canada; 18 Snyder Institute for Chronic Diseases, Faculty of Medicine, University of Calgary, Calgary, Alberta, T2N 4N1, Canada

**Keywords:** Physical fitness, Cerebrovascular function, Cognition, Aging

## Abstract

**Background:**

Aging and physical inactivity are associated with declines in some cognitive domains and cerebrovascular function, as well as an elevated risk of cerebrovascular disease and other morbidities. With the increase in the number of sedentary older Canadians, promoting healthy brain aging is becoming an increasingly important population health issue. Emerging research suggests that higher levels of physical fitness at any age are associated with better cognitive functioning and this may be mediated, at least in part, by improvements in cerebrovascular reserve. We are currently conducting a study to determine: if a structured 6-month aerobic exercise program is associated with improvements or maintenance of both cerebrovascular function and cognitive abilities in older individuals; and, the extent to which any changes seen persist 6 months after the completion of the structured exercise program.

**Methods/design:**

Two hundred and fifty men and women aged 55–80 years are being enrolled into an 18-month combined quasi-experimental and prospective cohort study. Participants are eligible for enrollment into the study if they are inactive (i.e., not participating in regular physical activity), non-smokers, have a body mass index <35.0 kg/m^2^, are free of significant cognitive impairment (defined as a Montreal Cognitive Assessment score of 24 or more), and do not have clinically significant cardiovascular, cerebrovascular disease, or chronic obstructive pulmonary airway disease. Repeated measurements are done during three sequential six-month phases: 1) pre-intervention; 2) aerobic exercise intervention; and 3) post-intervention. These outcomes include: cardiorespiratory fitness, resting cerebral blood flow, cerebrovascular reserve, and cognitive function.

**Discussion:**

This is the first study to our knowledge that will examine contemporaneously the effect of an exercise intervention on both cerebrovascular reserve and cognition in an older population. This study will further our understanding of whether cerebrovascular mechanisms might explain how exercise promotes healthy brain aging. In addition our study will address the potential of increasing physical activity to prevent age-associated cognitive decline.

## Background

Aging is associated with declines in certain cognitive domains and lower levels of physical activity [1,2]. The Public Health Agency of Canada [2] and World Health Organization [3] report that adults between 65 and 74 years of age are the most sedentary portion of the population. An estimated 60% of older adults lead sedentary lifestyles and do not engage in enough physical activity to achieve favorable health benefits [4]. Physical inactivity is a recognized modifiable risk factor of cerebrovascular disease and the cognitive decline seen in older adults [5,6]. With the projected doubling of the number of older Canadians over the next 25 years [7], it is anticipated that the personal and societal burden of age-associated conditions like cerebrovascular disease and dementia (e.g., Alzheimer’s disease, AD) will increase substantially [8].

Post-maturational aging is associated with reduced abilities in a number of cognitive processes, including attention, learning and memory, and executive control [9]. These age-related changes, while detectable, are generally considered part of the aging process and usually do not interfere with the ability to live independently. Dementia, an acquired decline in multiple cognitive areas causing a significant impairment in social or occupational functioning , occurs in approximately 8% in the older population, with AD and vascular dementia (VaD) either alone or in combination the most common causes [10,11]. As both aging and vascular disease are risk factors for both AD and VaD, age-associated declines in cerebrovascular function might contribute to the development of these disorders [11-13].

With normal aging there is an approximately 5% decrease per decade in resting cerebral blood flow (CBF) [14]. Although the brain represents 2.0 to 2.3% of an adult’s total body weight, in percentage terms it accounts for ten times more of the body’s total resting energy consumption [15,16]. Since cerebral tissue does not produce or store sources of energy, its high metabolic need requires constant and adequate blood supply. Age-related hypoperfusion may be associated with sufficient reductions in the delivery of oxygen and nutrients and inadequate removal of metabolic by-products to produce impaired cognitive function and slowly progressive cellular injury [17]. Neurons, glial and vascular cells are linked together in neurovascular units where neuronal and glial signals control local CBF [17-19]. With aging, neurovascular coupling is disrupted, which could result in the metabolic requirements of the brain tissue not being matched by CBF [15,18]. Decreases in CBF have been associated with a number of age-related neurodegenerative changes including a loss of neuropil (i.e., synaptically dense regions of the nervous system mainly composed of unmyelinated axons, dendrites and glial processes) [20].

There is consistent evidence that regular exercise promotes brain health and is associated with a lower risk for age-related cognitive decline and dementia [21], however the underlying mechanisms have not been well defined [22]. Neuroimaging studies of older adults indicate that higher levels of cardiovascular fitness are associated with larger volumes of specific brain regions including the hippocampus, an important region for learning and memory [23,24]. This effect may be attributable, in part, to neurogenesis [25] possibly mediated by increases in vascularization, elevated levels of neurotrophins and growth factors, and/or improved neuronal survival in the aging brain [26].

Previous studies have focused primarily on neuronal processes that may explain why physical activity promotes healthy brain aging while cerebrovascular mechanisms that might underlie improved cognitive function are relatively poorly understood. Pharmacological manipulation of cerebral blood flow can modify performance on a variety of cognitive tasks in both animal models and humans (for review see [15,27]). Cerebrovascular networks are thought to have a high degree of plasticity (i.e., they are changeable or modifiable) [27]. Exercise appears to stimulate the growth of new capillaries from preexisting vessels in the brain and improve resting CBF [27,28]. We recently demonstrated that increased levels of physical fitness were associated with greater cerebrovascular reserve (i.e., the ability of cerebral arteries to dilate in response to a stimulus) and improved cognitive function [12]. These data suggest that the link between physical fitness and cognition may be mediated, at least in part, by improvements in cerebrovascular function.

We are conducting a quasi-experimental prospective cohort study with two primary aims: 1) to determine the effect of a six-month aerobic exercise intervention on physical fitness, resting CBF, cerebrovascular reserve, and cognitive function in 250 sedentary men and women between the ages of 55 and 80 years; and, 2) to determine the extent to which the changes in cerebrovascular and cognitive function persist six months following the completion of the exercise intervention. We hypothesize the improvement in cognitive function derived from regular aerobic exercise is at least partially mediated by enhanced cerebrovascular reserve. Further, we hypothesize that these beneficial changes will persist in participants who remain physically active as compared to those who revert to a sedentary lifestyle. The purpose of this manuscript is to describe the study design and research methodologies in detail.

## Methods

**Figure 1:**
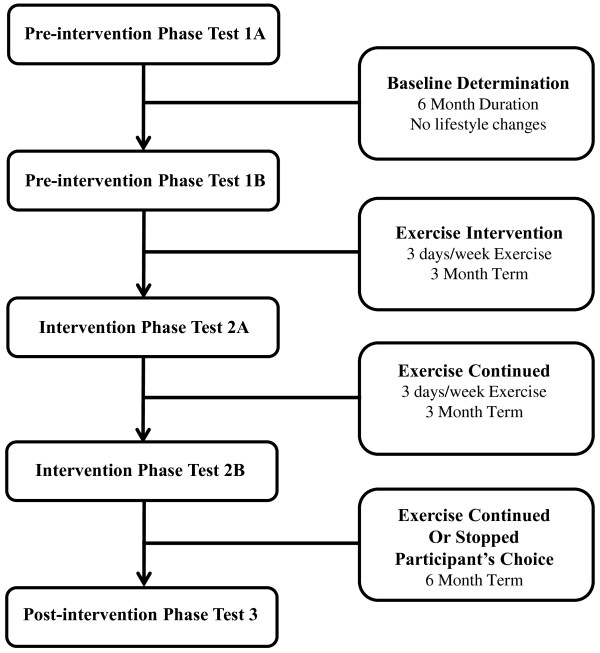
Flow chart of the phases of the BIM study.

The *Brain in Motion* (BIM) study is an 18-month combined quasi-experimental and prospective cohort study consisting of three six-month phases: 1) pre-intervention phase; 2) aerobic exercise intervention phase; and 3) post-intervention (Figure 1).

### Participant recruitment

The target population for enrollment is sedentary men and women aged 55–80 years. Participants are being recruited through media, poster and newspaper advertisements. Potential participants must provide informed written consent prior to enrollment into the study. Participants are also invited to provide separate written consent for genetic testing (see section on Blood Sampling and Biomarker Assessments). The study protocol was approved by the University of Calgary Conjoint Health Research Ethics Board.

#### Inclusion/exclusion criteria

During an initial phone interview, interested participants are assessed for their eligibility based on pre-determined inclusion and exclusion criteria (Table 1). Potential participants who successfully complete an initial telephone-based evaluation are then scheduled for a 60-minute on-site assessment of eligibility (Table 1).

**Table 1:** Inclusion criteria – participant must fulfill all criteria to be admitted to the study

**Assessment phase**	**Criteria**
Telephone interview	Participant engages in less than 30 minutes of moderate exercise four days per week or 20 continuous minutes of vigorous exercise two days per week
	Participant has a body mass index (BMI) of less than 35 kg/m^2^
	Participant is able to walk independently outside and up and down at least 20 stairs
	Participant has not been diagnosed with an active cardiovascular/cerebrovascular disease or obstructive airway disease that would preclude their ability to safely exercise
	Participant has been a non-smoker for at least 12 months
	Participant has not had major surgery or trauma in the last six months
	Participant is free of debilitating neurological disorders (e.g., Multiple Sclerosis and AD)
	Participant has written permission from their attending physician to participate in the study
On-site assessment	Montreal Cognitive Assessment (MoCA) score ≥ 24 [29]
	History (i.e., cardiovascular, respiratory, hepatic, endocrine, neurological, endocrine, and musculoskeletal health) and physical examination by a study physician to assess safety/appropriateness for exercise. Current medications (i.e., prescribed, over-the-counter, and complementary agents), allergies, and/or history of any substance abuse issues noted.

### Study design

The BIM study design and timeline is outlined in Figure 1. A second baseline measure allows an estimate of practice effects on the cognitive test performance and an estimate of the consistency of our other various measures. Additionally, this second assessment ensures feasibility of the testing in the selected study population and that participants will comply with follow-up testing prior to the exercise intervention.

#### Pre-intervention baseline phase test 1A (month 0)

The baseline assessment during the pre-intervention phase is conducted during four separate on-site appointments during which the following are done: 1) fasting blood work; 2) incremental treadmill test to assess maximal aerobic capacity (VO_2_max); 3) assessment of cerebrovascular function; and 4) assessment of cognitive function, mood and sleep (Table 2). In addition, participants are required to fill out a *Dietary History Questionnaire* based on their intake over the previous 12 months [30]. Details of these evaluations are given later.

**Table 2:** Table of testing and questionnaires at each phase

	***Time (months)***				
	**0**	**6**	**9**	**12**	**18**
	***Phase***				
**Tests**	**1A**	**1B**	**2A**	**2B**	**3**
Blood work	✓	✓	✓	✓	✓
Genetics	✓				
Maximal aerobic capacity	✓	✓	✓	✓	✓
Cerebrovascular function	✓	✓	✓	✓	✓
Cognitive function	✓	✓		✓	✓
**Questionnaires**					
Mood	✓	✓	✓	✓	✓
Sleep	✓	✓	✓	✓	✓
Lifetime physical activity		✓			
Past year physical activity				✓	
Past six month physical activity					✓
Diet History	✓			✓	
Social Support				✓	

#### Pre-intervention baseline phase test 1B (month 6)

Six months following enrollment into the study, and immediately prior to the exercise intervention phase, participants undergo the same evaluations as done during *Pre-Intervention Baseline Phase Test 1A* (see Table 2 for details). At an additional visit, the *Lifetime Physical Activity Questionnaire*[31] is administered by study personnel.

#### Exercise intervention phase (months 6–12)

Participants then take part in the supervised six-month aerobic training program that is held three days per week at the Fitness Centre in the Faculty of Kinesiology at the University of Calgary. Each exercise session includes a five-minute warm up, aerobic exercise, a five-minute cool down, and stretching. Exercise prescriptions follow American College of Sports Medicine guidelines [32]. As participants progress through the exercise intervention, the duration of aerobic exercise (in addition to warm up and cool down) increases from 20 to 40 minutes. Exercise intensity is determined based on individual VO_2_max. The intensity builds from 30–45% until 60–70% maximum heart rate reserve (HRR) is achieved. Participants wear a Polar® heart rate monitor at each session to ensure compliance to their target heart rate zones. Heart rate data are collected and stored during each exercise session and are then exported offline following the exercise session to analyze heart rates during the exercise session (Polar® Team^2^ system). Participants are considered compliant if they attend at least 85% of the total exercise sessions. If an exercise session is missed, participants are strongly encouraged to “make-up” the session independently and record the unsupervised exercise session in a personal workout logbook. In addition to “make-up” exercise sessions, participants are also required to record any additional independent unsupervised exercise sessions.

#### Intervention phase test 2A (month 9)

Midway (3 months) through the exercise program, participants undergo repeat testing (same evaluations done during *Phase Test 1A/1B* (see Table 2 for details)) to assess for changes in parameters over time. Based on their results, exercise prescriptions for participants may be adjusted. In addition, sleep and mood are assessed by self-report questionnaires.

#### Exercise intervention phase test 2B (month 12)

Repeat testing is again done at the end of the 6-month intervention (see Table 2 for details). The *Dietary History Questionnaire*[28] and a *Past Year Physical Activity Questionnaire*[33] (details given later) are self-administered questionnaires completed at this time point.

#### Post-intervention phase test 3 (month 18)

Six-months following the completion of the exercise intervention, participants are re-evaluated (see Table 2 for details). During this six-month period, participants independently decide whether or not they continue with an exercise program. A *Past Six-Month Physical Activity Questionnaire*[33] is completed by participants to determine the amount of physical activity they engaged in following the completion of the supervised exercise sessions. This questionnaire permits a categorization of participants by their activity levels to help assess the extent to which changes in cerebrovascular and cognitive function are maintained six-months following the exercise intervention.

### Blood sampling and biomarker assessments

Fasted venous blood samples are collected five times from each participant as shown in Figure 1. Sex steroid hormone status (estradiol, progesterone, testosterone, sex hormone binding globulin), lipids (cholesterol, high and low density lipoprotein, triglycerides), hematology (complete blood count), thyroid (thyroid stimulating hormone), renal (creatinine), hepatic (alanine aminotransferase and bilirubin), and markers of vascular function influenced by age and physical fitness [34] (i.e., oxidative and nitrative stress, antioxidant enzyme capacity, and products of nitric oxide metabolism) are being measured.

For participants who provide consent for genetic testing, blood samples are also collected for this purpose. This testing will include catechol-O-methyl-transferase, brain-derived neurotropic factor, and apolipoprotein E ε4 genotyping, which have previously been shown to influence cognitive performance [35-37]. In addition, vascular endothelial growth factor genotyping, which can be influenced by exercise presumably via vascular-mediated mechanisms, will also be done [38].

### Maximal aerobic capacity and anthropometric measurements

All participants undergo assessments of fitness level by a maximal aerobic capacity (VO_2_max) test and anthropometric measurements during each phase. Participants are instructed to refrain from vigorous exercise the day of testing, ingesting caffeine and/or alcohol six hours prior to testing and eating a heavy meal four hours prior to testing. Anthropometric measurements include height, weight, circumference measurements (waist and hip), body composition (bioelectrical impedance analysis), and grip strength. The VO_2_max test is conducted on a motorized treadmill and follows the Bruce protocol [39]. A plateau in oxygen uptake with increasing work rate is observed (<2 mL/kg/min), a respiratory exchange ratio (RER) of at least 1.15, and age-predicted maximal heart rate (210-(age X 0.65)) are used as criteria for stopping the test (as recommended by the Canadian Society for Exercise Physiology (CSEP) [40] and American Thoracic Society (ATS) [41]). Outcome measures for the VO_2_max test include oxygen uptake, carbon dioxide production, ventilation (tidal volume and breathing frequency), rating of perceived exertion (Borg scale), continuous heart rate recordings (12-lead electrocardiogram (ECG)), and systemic blood pressure.

This fitness test determines each study participant’s maximal aerobic capacity that is used to determine work rates for the submaximal exercise test (during the cerebrovascular function test) and the target heart rate zones for the exercise intervention.

### Cerebrovascular function

Cerebrovascular response to carbon dioxide and submaximal exercise is assessed in participants who have refrained from exercising the day of the test and eating or drinking anything other than water, two hours prior to testing. A capillary blood sample is taken from the middle finger and immediately analyzed for PO_2_ and PCO_2_, acid/base status, glucose, hematocrit, potassium, sodium, chloride, and calcium levels (Radiometer ABL 800, Denmark). Participants sit quietly in a chair while they are fitted with monitoring equipment. In order to noninvasively measure the CBF of the middle cerebral artery (MCA), a 2-MHz transcranial Doppler ultrasound (TCD) is used (Toc Neurovision™, Multigon Industries, Inc., Yonkers, NY) [42]. To locate the MCA, the TCD probe is placed in the temporal region just above the end of the zygomatic process close to the ear, using techniques previously described [42]. Maximum peak velocity ($\bar{V}P$), intensity-weighted mean ($\bar{V}\text{IWM}$), an index of the cross-sectional area of the vessel called power ($\bar{P}$), cerebrovascular conductance, and cerebrovascular reserve are recorded as previously described [12]. Outcome measures for this test include continuous TCD recordings, heart rate (3-lead ECG; Micromon 7142 B, Kontron Medical, Milton Keynes, UK), blood pressure (beat-by-beat using finger pulse photoplethysmography, Finometer, Finapres Medical Systems, Amsterdam, The Netherlands), and arterial hemoglobin saturation (finger pulse oximetry; 3900p, Datex-Ohmeda, Madison, WI, USA).

We have previously developed and tested the protocol for this test [12]. In short, baseline end-tidal respiratory measures (PCO_2_ and PO_2_) are recorded using dedicated software (Chamber, University Laboratory of Physiology, Oxford, UK), during a 10-minute baseline period. With their nose occluded, each participant breathes through a mouthpiece connected to a fine capillary that is connected to a mass spectrometer (AMIS 2000, Innovision, Odense, Denmark) in which end-tidal respiratory measures (PCO_2_ and PO_2_) are analyzed. These end-tidal gases are averaged over the 10 minutes, and are used to determine the desired end-tidal pressure carbon dioxide (PET_CO2_) and end-tidal pressure oxygen (PET_O2_) for cerebrovascular response to an euoxic hypercapnia test. Control of desired PET_CO2_ and PET_O2_ values is achieved accurately and continuously using sophisticated software (BreatheM v2.40, University Laboratory of Physiology, Oxford, UK) and dynamic end-tidal forcing technique as previously described [43,44]. The test lasts 17 minutes with two 3-minute steps of carbon dioxide. For the first minute, participants breathe room air only. This time period is followed by a five-minute baseline period in which the PET_CO2_ is held at +1.0 mmHg above the participants’ resting PET_CO2_ value. Following baseline, PET_CO2_ is increased to +5.0 mmHg above normal resting values and held for three minutes and then is increased to +8.0 mmHg and is again held for three minutes. Lastly, PET_CO2_ is dropped back down to +1.0 mmHg above resting values and held for the final five minutes of the test.

Following the hypercapnia test, participants rest for 20–30 minutes. Then, participants are seated on the recumbent cycle ergometer and cerebrovascular response to submaximal exercise is assessed. This test lasts 30 minutes during which participants complete two six-minute intervals of exercise as previously described [12].

### Assessment of cognition, mood, sleep, and social support

Participants undergo cognitive testing four times, at six month intervals, during the course of the study (see Table 3 for details of the specific standardized and experimental tests done). The tests were chosen to assess cognitive domains that had previously been shown to be affected by fitness training, with a special focus on executive functions, processing speed, and memory [21,27,45]. In addition, we will look for changes in mood [46] and sleep [47,48] using validated self-report questionnaires. Involvement in the study might modify the perception by participants of their observed social support (i.e., the feeling that one is care for, has assistance available from other people, and that one is part of a supportive social network). As higher levels of perceived social support could increase adherence to the exercise program and have an independent beneficial impact on both mental and physical health (see [49] for review and discussion), perceived social support will be measured during the course of the study by a modified version of the Lubben Social Network Scale [50]. The testing sessions lasts approximately 120 minutes with short breaks taken as necessary. The same testing order is maintained through all assessments during the study.

**Table 3:** BIM neuropsychological assessment

**Domain**	**Test**	**Description**
Attention	Attention Network Test [51-53]	Computerized test of the efficiency of 3 attention networks, including tests of altering, orienting and executive control (flanker test)
	Auditory Consonant Trigram Test [54]	Auditory presentation of 3 consonant trigrams (i.e., three single consonant letters) followed by a number. The subject is instructed to subtract from that number for several seconds until cued, and then asked to recall the letters
Processing Speed	Symbol-Digit Modalities [55]	Simple substitution task pairing specific numbers with given geometric figures. Both written (90 seconds) and oral (90 seconds) forms administered
Verbal Memory	Buschke Selective Reminding Test [56,57]	1. Memory Test (List learning of 12 words rehearsed for six trials)
		2. Cued Recall
		3. Multiple Choice recognition
		4. Oral delayed recall of 12 words (30 minutes following multiple choice)
Visual Memory	Medical College of Georgia Complex Figures Test (MCG) [58]	A test of visual memory in which the subject is asked to copy the figure and then draws it from memory at an immediate test and at a 30 minute delay
Executive Function	Delis-Kaplan Executive Function System (D-KEFS) [59]	1. Card Sorting Test- one card set is randomly assigned to each session (i.e., one card set per phase) in which the free-sorting and recognition conditions are tested
		2. Color-word Interference Test- four conditions are presented to participants (i.e., colour naming, word reading, inhibition, and inhibition/switching) and participants must follow the presented rule to orally complete the task as accurately as possible
		3. Verbal Fluency Test- participants must say as many words as possible in 60 seconds for each condition (i.e., letter fluency, category fluency, and category switching)
Premorbid Intellectual Ability	North American Adult Reading Test [60]	61 different rare words are presented with scoring based on American and Canadian punctuation rules
Mood	Profile of Mood States [46,61]	65 adjectives are presented that are rated by subjects using a 5-point scale. From the responses 6 factors are derived (tension-anxiety, vigour-activity, depression-dejection, fatigue-inertia, anger-hostility, and confusion-bewilderment
Sleep	Pittsburg Sleep Quality Index [47]	Self-report questionnaire of the last month sleep habits/sleep quality
	Epworth Sleepiness Scale [48]	Self-report of general daytime sleepiness
Social Support	Modified version of the Lubben Social Network Scale [50]	Self-report questionnaire of perceived social support from family, friends, and other study participants
Cognitive Activity	Lifetime Cognitive Questionnaire (*Test 1A* only) [62]	20 questions that assess cognitive activities participants were engaged in during childhood (age 6), teens (age 12), young adults (age 18), and adulthood (age 40)
	Current Cognitive Questionnaire (all test sessions) [62]	23 questions that assess the degree to which participants are cognitively active in their lives at the present time

### Questionnaires

#### Physical activity questionnaires

At *Pre-intervention Phase Test 1B* participants complete the interviewer-administered *Lifetime Physical Activity Questionnaire* to quantify lifetime occupational, household, and exercise/sports activities and used to determine lifetime physical activity patterns [31]. The *Past Year Physical Activity Questionnaire*[33] is self-reported and given to participants immediately following the completion of the exercise intervention, which assesses physical activities while enrolled in the BIM study. At the completion of the study (*Post-intervention Phase 3*), participants are given the *Past Six Month Physical Activity Questionnaire* to assess their activity level six-months following completion of the exercise intervention.

#### Diet history questionnaire

The *Diet History Questionnaire* (DHQ) is a self-administered food frequency questionnaire originally designed by the National Cancer Institute but modified for use in Canada [30]. Consisting of 146 questions about 124 different food items, the questionnaire includes questions about portion size, seasonal intake, and fat usage within the past 12 months [30]. Participants complete the questionnaire at enrollment into the study and following the completion of the exercise intervention.

### Statistical analysis

The primary outcome measure is the change in overall cognitive function (defined as the average of standardized cognitive domain scores) over time. The sample size estimates assume a two-tailed alpha level of 0.05, power of 90% (β = 0.1) and a change in overall cognitive score of 0.2 standard deviation units based on Brown et al. [12]. To account for a predicted 15% dropout rate to account for withdrawals [63] and non-compliance with the exercise sessions (i.e., attendance 85% or less of all sessions), we will recruit 125 men and 125 women (n = 250). The primary outcome analysis within individuals will use repeated measures analysis of variance using a general linear mixed model. Cardiorespiratory fitness, vascular measurements of resting CBF, cerebrovascular reserve, cerebrovascular conductance, mood and sleep are secondary outcome measures. The covariates being considered in our analysis include age, sex, education, socio-economic status, BMI, hormones, lipids profiles, genetics, diet, physical activity and physical fitness (i.e. VO_2_max). Multivariable methods will be used to determine if covariates are predictors of our primary outcome measure (overall cognition) [12].

## Discussion

Current projections suggest that by 2031, approximately 25% of the Canadian population will be over the age of 65 [7] and in 2050, the estimated world population over the age of 60 will be 2 billion [3]. With societal aging, a progressive increase in the absolute number of individuals suffering from age-associated health issues like cerebrovascular disease, VaD, and AD will inevitably occur unless we can change the likelihood of these events occurring. If we cannot, population aging will lead to a significantly greater burden on the health and social care system of our country and around the world. Effective prevention of these diseases is of paramount importance. A promising approach is encouraging higher levels of physical activity since exercise may delay or prevent the progression of cognitive decline associated with aging, prevent cerebrovascular disease, and delay the onset of AD and related dementias [64].

The BIM study is designed to add to the literature on whether exercise can be an effective means of decreasing the effects of aging on cognition. This study will combine detailed physiological and cognitive data with sociodemographic, biologic, and lifestyle information obtained from a relatively large and well defined older population. By performing an exercise intervention we are able to investigate the extent to which cerebrovascular physiology might act as a mediating factor between exercise and cognition. If confirmed this raises the possibility of cerebrovascular measures functioning as a surrogate marker of brain health in future studies of exercise interventions.

The BIM study uses state-of-the-art measurements of cerebrovascular function, physical fitness, cognitive function, lifestyle, sleep, biologic, and genetic markers all of which strengthen the study. The Laboratory for Human Cerebrovascular Physiology houses sophisticated equipment to control end-tidal PO_2_ and PCO_2_ accurately and continuously using our dynamic forcing-function system. The VO_2_max testing protocols used in the study are considered the “gold-standard” practice for the assessment of physical fitness, thereby adding strength to the study since we avoid problems associated with predictive tests by measuring VO_2_max rather than VO_2_peak [65]. The comprehensive neuropsychological test battery allows us to assess multiple aspects of cognitive function. The battery is administered at multiple time points through the study that will allow the assessment of whether the exercise intervention has an impact on cognitive function, how long it takes to become evident, and its duration. We have included numerous self-administered questionnaires to measure changes in dietary intake, sleep habits, physical activity levels and cognitive abilities at differing time points. Collectively, these data will provide insights regarding the possible mechanisms whereby exercise improves cerebrovascular and cognitive function.

The current study is unique because all the participants of our study attend our supervised and structured exercise program for six-months. Further, the exercise program is individually tailored to each participant as exercise prescriptions are based on each individual’s heart rate reserve. This supervised intervention assesses participants’ progression through the exercise program with modifications to the intervention made as needed. The BIM study uses a quasi-experimental prospective cohort design because it reduces variability over time since each participant is their own control during the repeated baseline assessment over a six-month period. An inherent weakness is the potential carry over or practice effects in repeated cognitive testing. We have attempted to deal with this issue by 1) repeating the baseline cognitive testing to observe the stability of the performance measures over time before the intervention, 2) using alternate forms of tests when possible (i.e., verbal fluency, selective reminding, MCG figure, card sorting), and 3) we will analyze the cognitive data for overall trends at the four time points to examine for a global practice effect over time. The next logical step in our research program will be a randomized controlled trial in first a healthy and then in a diseased population (e.g. individuals with Mild Cognitive Impairment or stroke).

The mechanisms whereby exercise-induced increases in cerebrovascular reserve improve cognitive function are unknown. At this time, we are focused on demonstrating the physiologic mechanisms by which exercise confers its beneficial influence on cognition. The BIM study is the first to examine the influence of an aerobic exercise intervention on cerebrovascular and cognitive function in a healthy aging population. The results from this study may inform the development of new therapies to reduce or prevent the cognitive decline associated with aging.

## Abbreviations

AD: Alzheimer’s disease; CBF: Cerebral blood flow; BIM: Brain in motion; BMI: Body mass index; MoCA: Montreal cognitive assessment; VO_2_max: Maximal aerobic capacity; HRR: Heart rate reserve; RER: Respiratory exchange ratio; ECG: Electrocardiogram; MCA: Middle cerebral artery; PO2: Endtidal oxygen; PCO2: Endtidal carbon dioxide; TCD: Transcranial doppler ultrasound; VaD: Vascular dementia; $\bar{V}P$: Peak velocity; $\bar{V}\text{IWM}$: Intensity-weighted mean; $\bar{P}$: Power; PET_CO2_: End-tidal pressure carbon dioxide; PET_O2_: End-tidal pressure oxygen; D-KEFS: Delis-kaplan executive function system; MCG: Medical college of georgia complex figure; DHQ: Diet history questionnaire.

## Competing interests

The authors declare that they have no competing interests.

## Authors’ contributions

GAE, CMF, MDH, DBH, RSL, TJA, RL, EES, MJP have made substantial contributions to conception and design of this study. AVT and MHD wrote the draft of the manuscript and all authors were involved in revising the manuscript critically for important intellectual content. BJW provided significant medical coverage for exercise testing. GMB and EH were involved in coordinating the study and recruitment. GAL contributed the social support questionnaire to the study. All authors have given final approval of the version to be published.

## Pre-publication history

The pre-publication history for this paper can be accessed here:

http://www.biomedcentral.com/1471-2318/13/21/prepub
